# Olfactory Cleft Measurements and COVID-19–Related Anosmia

**DOI:** 10.1177/0194599820965920

**Published:** 2020-10-13

**Authors:** Aytug Altundag, Duzgun Yıldırım, Deniz Esin Tekcan Sanli, Melih Cayonu, Sedat Giray Kandemirli, Ahmet Necati Sanli, Ozge Arici Duz, Ozlem Saatci

**Affiliations:** 1 Department of Otorhinolaryngology, Medical Faculty, Biruni University, Istanbul, Turkey; 2 Department of Ear, Nose, and Throat, Acibadem Taksim Hospital, Istanbul, Turkey; 3 Vocational School of Health, Mehmet Ali Aydınlar University, Istanbul, Turkey; 4 Department of Radiology, Acibadem Taksim Hospital, Istanbul, Turkey; 5 Department of Radiology, Acibadem Kozyatagi Hospital, Istanbul, Turkey; 6 Department of Otorhinolaryngology and Head & NeckSurgery, Ankara City Hospital, Ankara, Turkey; 7 Department of Radiology, University of Iowa, Iowa City, Iowa, USA; 8 Department of General Surgery, Cerrahpasa Medical Faculty, Istanbul University-Cerrahpasa, Istanbul, Turkey; 9 Department of Neurology, Istanbul Medipol University, Istanbul, Turkey; 10 Department of Ear, Nose, and Throat, Sancaktepe Training and Research Hospital, Istanbul, Turkey

**Keywords:** SARS-CoV-2, olfactory cleft, width, volume, anosmia, Sniffin’ Sticks, COVID-19

## Abstract

**Objective:**

This study aimed to investigate the differences in olfactory cleft (OC) morphology in coronavirus disease 2019 (COVID-19) anosmia compared to control subjects and postviral anosmia related to infection other than severe acute respiratory syndrome coronavirus 2 (SARS-CoV-2).

**Study Design:**

Prospective.

**Setting:**

This study comprises 91 cases, including 24 cases with anosmia due to SARS-CoV-2, 38 patients with olfactory dysfunction (OD) due to viral infection other than SARS-CoV-2, and a control group of 29 normosmic cases.

**Methods:**

All cases had paranasal sinus computed tomography (CT), and cases with OD had magnetic resonance imaging (MRI) dedicated to the olfactory nerve. The OC width and volumes were measured on CT, and T2-weighted signal intensity (SI), olfactory bulb volumes, and olfactory sulcus depths were assessed on MRI.

**Results:**

This study showed 3 major findings: the right and left OC widths were significantly wider in anosmic patients due to SARS-CoV-2 (group 1) or OD due to non–SARS-CoV-2 viral infection (group 2) when compared to healthy controls. OC volumes were significantly higher in group 1 or 2 than in healthy controls, and T2 SI of OC area was higher in groups 1 and 2 than in healthy controls. There was no significant difference in olfactory bulb volumes and olfactory sulcus depths on MRI among groups 1 and 2.

**Conclusion:**

In this study, patients with COVID-19 anosmia had higher OC widths and volumes compared to control subjects. In addition, there was higher T2 SI of the olfactory bulb in COVID-19 anosmia compared to control subjects, suggesting underlying inflammatory changes. There was a significant negative correlation between these morphological findings and threshold discrimination identification scores.

**Level of Evidence:**

Level 4.

Olfactory dysfunction (OD) is a commonly recognized symptom of coronavirus disease 2019 (COVID-19).^1,2^ OD has a sudden onset, may be accompanied by taste disturbances, and can vary in severity ranging from hyposmia to anosmia.^3-6^ OD can have a qualitative effect, such as parosmia or phantosmia, or quantitative effects like hyposmia or anosmia.^6,7^

Postviral or postinfectious olfactory loss (PIOL) is characterized by a sudden loss of olfactory function after an upper respiratory infection (URI). Although OD is related to nasal mucosal swelling and secretions resulting in conductive blockage to the olfactory cleft (OC) region during a viral URI, sudden-onset anosmia related to COVID-19 is seen even in patients without nasal discharge or congestion.^5,8^

Several pathological mechanisms have been described for COVID-19 anosmia, including nasal cytokine storms and neurological tropism.^9^ The most widely accepted potential mechanism involves the nasal cavity being the possible viral entrance site for initial infection with severe acute respiratory syndrome coronavirus 2 (SARS-CoV-2) and also the dominant replication site.^10^

The authors of the current study had previously shown increased OC width and volume in PIOL patients compared to control subjects.^11^ Based on that study, we speculated that a wider OC than normal was a risk factor for a variety of chemical and biological factors, such as air pollutants and viral infections.^11^ Based on our previous experience on postviral anosmia and increased OC width/volume compared to controls, we aimed to evaluate whether such a relationship exists for COVID-19 anosmia and whether there is any difference in OC measurements between postviral anosmia and COVID-19 anosmia.

In this study, we aimed to investigate the OC region in terms of width and volume in patients with SARS-CoV-2 infection. This patient population was compared to patients with non–SARS-CoV-2–related OD and controls without OD.

## Materials and Methods

### Patient Selection

This study consisted of 91 individuals: 24 patients who were anosmic due to SARS-CoV-2, 38 patients with OD due to non–SARS-CoV-2 viral infection, and a control group of 29 normosmic cases who underwent paranasal sinus computed tomography (CT) to evaluate headache or tinnitus. Part of group 2 patient data was previously used as part of the study by our group.^11^ Control subjects were collected from a new patient pool.

The groups are defined as follows: group 1, anosmic patients due to SARS-CoV-2; group 2, anosmic patients due to non–SARS-CoV-2 viral infection; and group 3, healthy controls in terms of olfaction.

### Inclusion and Exclusion Criteria

Group 1 included patients with COVID-19 infection and subsequent anosmia. SARS-CoV-2 infection was confirmed with a positive reverse transcription polymerase chain reaction (RT-PCR) test from nasal and nasopharyngeal swabs based on World Health Organization (WHO) recommendations. COVID-19–related OD was assessed based on patient history and a 4-item odor identification test. After the conversion of the RT-PCR test to negative (average 3 weeks later), patients were evaluated with the Sniffin’ Sticks olfactory test.

Inclusion criteria for group 2 were history of a URI immediately prior to the olfactory loss, olfactory loss persisting for at least 8 weeks, pathological findings on the olfactory test (Sniffin’ Sticks olfactory test), no history of trauma or sinonasal surgery, no evidence of sinonasal inflammation on nasal endoscopy or paranasal sinus CT scan, and the existence of CT scan images at the time of olfactory evaluation. The patients in group 2 included patients evaluated between 2015 and 2019.

Exclusion criteria for groups 1 and 2 were age younger than 18 years, pregnancy, normosmia detected on Sniffin’ Sticks olfactory test (a threshold discrimination identification [TDI] score of >30.5), acute and/or chronic rhinosinusitis or other acute/chronic nasal disease, nasal polyposis, allergic or idiopathic rhinitis, posttraumatic olfactory loss, severe turbinate hypertrophy or nasal septum deviation affecting the air passage, malignancy history, and a history of nasal or paranasal surgery.

Group 3 was selected from the patients who underwent paranasal sinus CT due to headache or tinnitus and Sniffin’ Sticks olfactory test available. These cases were normosmic according to these test results. Patients with severe nasal septal deviation and turbinate hypertrophy affecting the olfactory area at a level that prevents air passage were not included in the control group.

### Ethics

The study protocol was approved by the medical research ethics committee of Istanbul Medipol University (495/10.06.2020). The study complied with the Declaration of Helsinki. Written informed consent was obtained from all participants.

### Olfactory Examination

#### Four-item odor identification test

OD in COVID-19 patients was initially assessed using a 4-item odor identification test. The test consists of 4 bottoms, including rose, clove, orange, and mint. Using a multiple-choice paradigm, patients were asked to find the correct odor descriptions from a verbal list of 4 descriptors each. A score of 0 was accepted as anosmia in a 4-item odor identification test. This test was applied only to group 1.

#### Sniffin’ Sticks olfactory test

Olfactory testing was performed using the Sniffin’ Sticks test (Burghart GmbH).^12-15^ The olfactory test was performed for all participants. The patients with COVID-19 infection included in group 1 were evaluated with the Sniffin’ Sticks olfactory test after conversion of the RT-PCR test to negative. The mean interval between Sniffin’ Sticks tests from the first onset of anosmia complaints was 22 days (14-30 days) in group 1 and approximately 4 months in group 2. Odorants were presented in felt-tip pens. For odor presentation, the investigator first removed the cap and then placed the tip of the pen in close proximity to the subject’s nostrils. Olfactory function was evaluated in terms of odor threshold, odor discrimination, and odor identification. The clinical evaluation of olfactory performance was based upon a composite of the TDI score represented by sum of the scores from 3 subsets. A TDI score below 16.5 was accepted as functional anosmia.^12-15^ The applicability of the Sniffin’ Sticks test for the target population has been previously validated.^16^

### OC Measurements

#### CT technique

Patients in groups 1 and 2 underwent CT to rule out underlying organic or obstructive sinus pathologies. Patients in group 3 had paranasal CT images taken for headache and tinnitus.

In order to keep the X-ray exposure low, a narrow-window paranasal sinus CT was acquired, including the OC. All CT exams were performed with a 128 × 2-slice dual-source CT scanner (Siemens; Flash Definition). After screening of the paranasal sinus region using 0.625-mm collimation, the OC region was reformatted at a 0.4-mm section thickness and 0.1-mm increment with a centralized smaller field of view (FOV). Aeration of OC was assessed by creating sharp-edge (bone kernels) reconstructions on the coronal plane.

**Figure 1.**
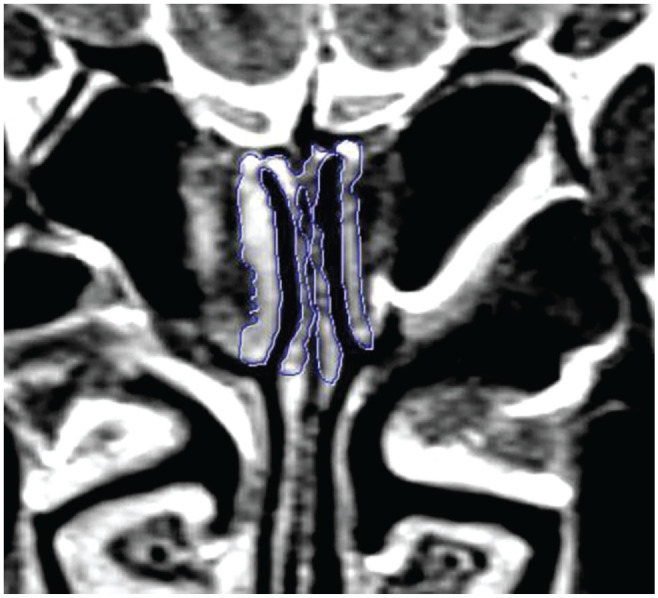
Section through the parasagittal plane of the cribriform plate: cross section from the front one-third to the back two-thirds section perpendicular to the horizontal plane, the plan in which the coronal plan image was obtained.

CT measurements were performed with a special workstation that allows very precise digital measurements (Syngo.Via Software VB30A; Siemens). The boundaries of OC were determined using successive coronal plane sections of 1 mm. The anterior boundary was defined as the anterior attachment of the middle turbinate since the vertical lamella of middle concha is usually not deformed and has a vertical course without much deviation. Since the volumetric analysis was performed with these predefined planes in all patient populations, the chosen landmarks would not have an effect on differences among the groups. The posterior boundary was defined as the anterior wall of the sphenoid sinus. The medial boundary was the nasal septum, and the lateral boundary was the middle and superior turbinates.^17^ On CT, OC diameters were measured in the coronal plane perpendicular to the horizontal plane passing through the anterior one-third and the posterior two-thirds intersection point, which could be evaluated more easily since the aeration was always preserved (**Figure 1**). In this coronal section, based on a plane parallel to the cribriform platform, a region up to 10 mm deep from the roof of the cleft was taken into account, and an appropriate voxel-of-interest (VOI) plan was measured by making manual free VOI drawings. For the volume calculation, we used a practical and easy special software that directly measures the volume of the OC based on the segmentation of landmarks instead of measuring separately width, depth, and length to reduce operator-dependent exaggerations or miscalculations.

**Figure 2.**
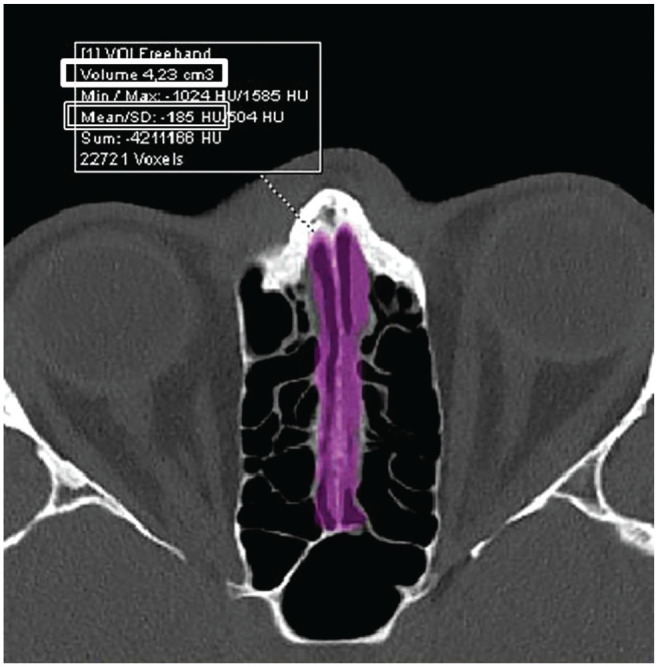
Axial image depicting the total volume of the olfactory cleft (thick-edged rectangle, cm^3^) and mean density within the voxel of interest (thin double-line edged rectangle).

Calculated volumes were recorded in cm^3^ and the average density of the total volume within the voxel was recorded in Hounsfield units (**Figure 2**). The olfactory fossa depth was checked based on the position of the cribriform plate relative to the ethmoid roof based on the Keros classification.^18^

#### Magnetic resonance imaging technique

**Figure 3.**
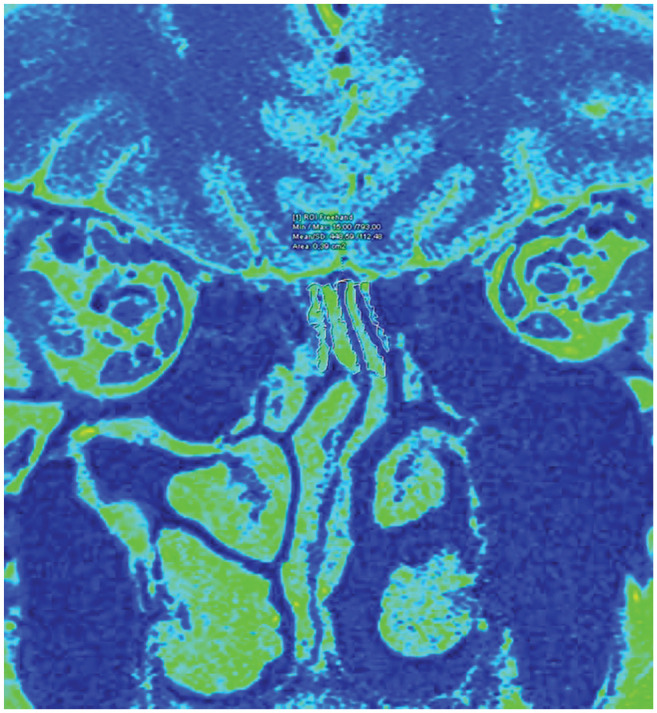
Grayscale and color window coronal T2 cleft magnetic resonance image. The mean region of interest signal intensity value was measured by taking the olfactory cleft mucosa along the height extending from the cleft top to 10 mm inferior.

All group 1 to 2 patients in the study had a multiparametric odor functional magnetic resonance imaging (MRI) assessment in our unit that is specialized on smell-taste disorders and has the latest technological equipment. Group 3 patients had auditory functional magnetic resonance examinations. MRI scanning was performed on a 3 Tesla MRI unit (3 Tesla Magnetom MRI unit; Siemens). For OC imaging, a 32-channel head coil was used. After the localizing images were obtained, thin-section ultra-high-resolution coronal T2 images (TR: 6550 ms; TE: 99 ms; flip angle: 150°, slice thickness: 1 mm; distance factor: 0; FOV: 100 × 100 mm; matrix: 269 × 384; phase oversampling: 56%; bandwidth: 289 Hz/pixel; voxel size: .6 × .6 × .6 mm; time of acquisition: 8.19 minutes; turbo factor: 17) extending from the anterior pole of the olfactory bulb to the primary olfactory region were obtained. The coronal line detected by CT was interpolated to the MRI sections, and the OC mucosa was assessed by defining regions of interest (ROIs) up to a depth of 10 mm from the cribriform plate roof. Only the mucosa was delineated without including the hypointense line of the bony periosteum. T2 signal brightness was taken into account radiologically in terms of showing a more quantitative effect of mucosal inflammation-edema. T2 signal intensity (SI) was measured by placing predefined ROIs on magnified images. The average T2 SI was recorded for all patients (**Figure 3**). In the indicated area along with the OC, in addition, olfactory bulb volumes (OBV) and olfactory sulcus (OS) depths were also measured on fluid-attenuated inversion recovery (FLAIR) sequences on the same coronal plane on MRI for group 1 and 2 patients.

All patients underwent CT and MRI within an average of 7 days after the Sniffin’ Sticks test. All measurements were performed by a single radiologist experienced in head and neck radiology. The radiologist was blind to the demographic and clinical information of the patients.

### Statistical Analysis

The statistical analysis was performed using SPSS for Windows, version 17.0 software (SPSS). Descriptive statistics were expressed as numbers and percentages for categorical variables and as mean, standard deviation, median, and interquartile range for numerical variables. An analysis of variance (ANOVA) with post hoc Dunnett’s test was used to compare normally distributed variables between 3 independent groups. Student *t* test was used to compare normally distributed quantitative variables between 2 groups. The relationship between numerical variables was analyzed using Pearson’s correlation analysis. A *P* value of <.05 was considered statistically significant.

## Results

The mean age of the total of 91 cases included in the present study was 39.3 ± 12 years. Group 1 (anosmic patients due to SARS-CoV-2) were younger compared to group 2 cases (anosmic patients due to non–SARS-CoV-2 viral infection) (*P* = .023). There was no significant difference between the groups in terms of sex (*P* = .56). The demographic features of the study groups are shown in **Table 1**.

**Table 1.** Demographic Features and Olfactory Test Results of the Study Groups.

Characteristic	Group 1 (n = 24)	Group 2 (n = 38)	Group 3 (n = 29)	*P* value
Age	35 ± 11.5	43.7 ± 11.8	36.9 ± 11	.009^a^
Sex (female/male)	14/10	21/17	13/16	.56^b^
Threshold	1.2 ± 0.5	1.7 ± 2	11 ± 1	<.001^a^
Discrimination	0.9 ± 1	1.5 ± 1.7	12.5 ± 1.5	<.001^a^
Identification	1.4 ± 2	2.3 ± 2.7	13.3 ± 0.7	<.001^a^
TDI scores^c^	3.6 ± 3.3	5.5 ± 5.1	35 ± 2.3	<.001^a^

Abbreviation: TDI, threshold discrimination identification.

a Analysis of variance with post hoc Dunnett’s test.

b Chi-square test.

c Sum of odor threshold, odor discrimination, and odor identification scores.

The mean interval between Sniffin’ Sticks tests from the first onset of anosmia complaints was 22 days (14-30 days) in group 1 and nearly 4 months in group 2. Based on the Sniffin’ Sticks test, all patients in groups 1 and 2 were anosmic, and all patients in group 3 were normosmic. Group 1 patients had a mean threshold (t) value of 1.2 ± 0.5, discrimination (d) of 0.9 ± 1, identification (i) of 1.4 ± 2, and total TDI score of 3.6 ± 3.3. The mean t value of group 2 patients was 1.7 ± 2; d value, 1.5 ± 1.7; i value, 2.3 ± 2.7; and TDI scores, 5.5 ± 5.1. Group 3 cases had a mean t value of 11 ± 1, d value of 12.5 ± 1.5, i value of 13.3 ± 0.7, and total TDI score of 35 ± 2.3 (**Table 1**).

The paranasal sinus CT and MRI scans of all groups were reviewed. The width of the right and left OC in groups 1 and 2 was significantly increased when compared to healthy controls (*P* < .001 for both). There was no significant difference between groups 1 and 2 in terms of OC width (*P* = .69 for the right OC and *P* = .61 for the left OC).

When total OC volume was compared between the groups, total OC volumes were found to be significantly higher in groups 1 and 2 when compared to the healthy controls (*P* < .001). However, there was no significant difference between groups 1 and 2 in OC volume (*P* = .36).

Another parameter investigated was the T2 SI of the OC area. The T2 SI of OC area in groups 1 and 2 was significantly higher when compared to healthy controls (*P* = .001), but there was no significant difference between groups 1 and 2 in terms of the T2 SI (*P* = .9). Pearson correlation showed that TDI scores and T2 SI of the OC area had a significant negative correlation (*P* < .001, *r* = −0.4).

**Figure 4.**
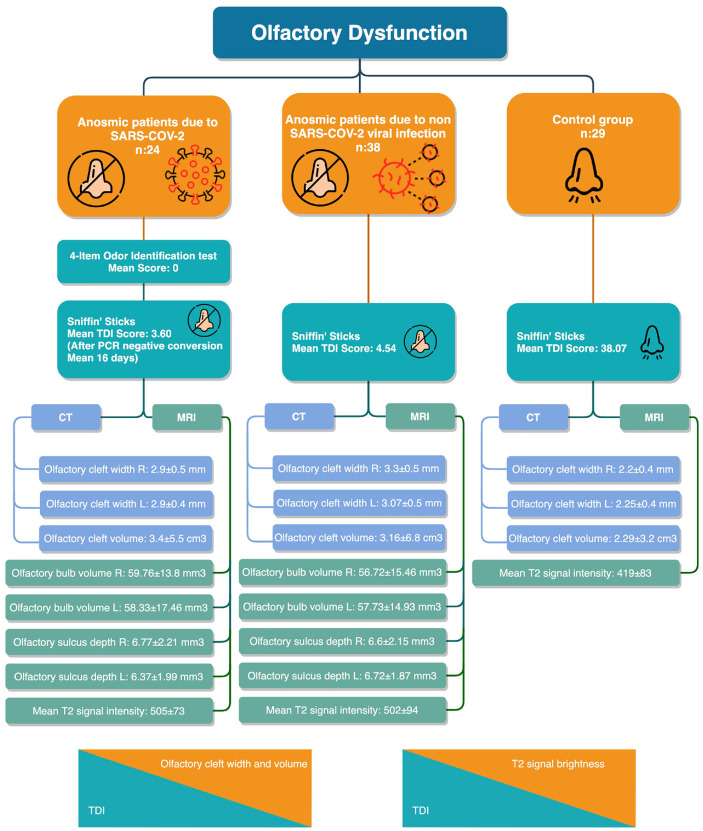
Flowchart of the findings.

The mean right OBV of group 1 cases was 59.76 ± 13.8 mm^3^, and the left OBV was 58.33 ± 17.46 mm^3^. The mean right OBV of group 2 cases was 56.72 ± 15.46 mm^3^, and the left OBV was 57.73 ± 14.93 mm^3^. The mean depth in group 1 cases was 6.77 ± 2.21 mm for the right OS and 6.37 ± 1.99 mm for the left OS; in group 2 cases, the depth was measured as 6.6 ± 2.15 mm for the right OS and 6.72 ± 1.87 mm for the left OS. There was no significant difference between the groups in OBV and OS depth (*P* = .436, *P* = .885, *P* = .770, *P* = .478, respectively) (**Table 2**). The findings of the study are summarized in **Figure 4**.

**Table 2.** Olfactory Cleft Width and Volume According to the Study Groups.

Characteristic	Group 1 (n = 24)	Group 2 (n = 38)	Group 3 (n = 29)	*P* value
Right olfactory cleft width, mm	2.9 ± 0.5	3.3 ± 0.5	2.2 ± 0.4	<.001^a^
Left olfactory cleft width, mm	2.9 ± 0.4	3.07 ± 0.5	2.25 ± 0.4	<.001^a^
Total olfactory cleft volume, cm^3^	3.4 ± 5.5	3.16 ± 6.8	2.29 ± 3.2	<.001^a^
Mean T2 signal intensity of olfactory cleft region	505 ± 73	502 ± 94	419 ± 83	<.001^a^
Right olfactory bulb volume, mm^3^				
Minimum-maximum (median)	39.4-82.1 (56.75)	28.9-100.3 (57.2)		.436^b^
Mean ± SD	59.76 ± 13.80	56.72 ± 15.46		
Left olfactory bulb volume, mm^3^				
Minimum-maximum (median)	20.9-91.2 (56.1)	29.8-104.6 (56.5)		.885^b^
Mean ± SD	58.33 ± 17.46	57.73 ± 14.93		
Right sulcus depth, mm				
Minimum-maximum (median)	2.5-10 (7.35)	3.1-10 (6.3)		.770^b^
Mean ± SD	6.77 ± 2.21	6.60 ± 2.15		
Left sulcus depth, mm				
Minimum-maximum (median)	2.7-9.7 (6.2)	4.1-10 (6.40)		.478^b^
Mean ± SD	6.37 ± 1.99	6.72 ± 1.87		

a Analysis of variance with post hoc Dunnett’s test.

b Student *t* test.

## Discussion

The major findings of this study were as follows: (1) total OC width was significantly wider in anosmic patients due to SARS-CoV-2 (group 1) and in anosmic patients due to non–SARS-CoV-2 viral infection (group 2) when compared to healthy controls; (2) similarly, OC volume was significantly higher in groups 1 and 2 than in healthy controls; and (3) T2 SI of OC mucosa (an indicator of inflammation) was higher in groups 1 and 2 than in healthy controls. T2 SI showed a negative correlation with TDI scores of Sniffin’ Sticks olfactory testing.

Angiotensin-converting enzyme 2 receptor (ACE2) and transmembrane protease serine 2 (TMPRSS2) intensity in the OC area, the most important transition point of the nose-to-brain pathway, have been demonstrated in previous studies.^19-21^ The high incidence of ACE2 and TMPRSS2 receptors in OC may explain the high affinity of the SARS-CoV-2 virus, which uses the same receptor.^22,23^ In recent studies, it has been suggested that ACE2 expression is higher in the sustentacular cells in the olfactory mucosa, except for the olfactory receptor, and SARS-CoV-2 mainly causes anosmia by supportive cell damaging.^24,25^ The underlying predisposition for increased OC volume in anosmic patients might be related to the overall increased ACE2 receptor number with the increasing olfactory mucosal surface area. Consequently, the probability of virus adherence may increase as well. However, in our study, we did not evaluate this histologically to confirm this hypothesis with histochemical measurements or virus testing.

In a recent study, increased OC width and volume were found to be associated with increased risk of PIOL. In that study, OC measurements of patients with PIOL were compared with healthy controls, similar in age and sex.^11^ Our study shows similar results, with OC width and volume significantly higher in anosmic patients due to SARS-CoV-2 just like in patients with OD due to non–SARS-CoV-2 viral infection.

Rapid immune response and “nasal cytokine storm” developed against intense viral involvement may be the main cause of sudden anosmia occurring in COVID-19.^26^ Wider OC and larger olfactory volume may result in more prompt nasal immune responses and/or rapid and intensive access to the olfactory bulb (OB). This supports the observation of increased frequent anosmia with large olfactory volumes due to a higher immunological response.

In our study, there was no significant difference in OB volumes between the COVID-19–related anosmia group and the postviral anosmia group. However, in the studies conducted so far, it has been shown that the OB volumes decrease due to olfactory receptor damage in postviral anosmia.^27,28^ In a study by Laurendon et al,^29^ OB volumes increased secondary to inflammation and edema in anosmia associated with COVID-19. In the same study, it was shown that volumes and signal intensities returned to normal on the 24th day. On the other hand, it was reported that there was no significant OBV and signal change in COVID-19–related anosmia. T2 signal brightness, which is evaluated as an indirect finding of inflammation and edema in MRI, was found to be higher in both cases with COVID-19–related anosmia and in the postviral anosmia group compared to the control group.^30^ This may be related to the fact that neuropathic damage is also experienced in the olfactory bulb, and at the same time, the virus creates neuropathic damage through the olfactory pathway in cases with symptomatic acute COVID-19–associated anosmia, as shown in other similar studies.^21,31,32^

Our study included the highest number of COVID-19–related anosmia cases who had both paranasal sinus CT and functional MRI among the studies so far. Another major strength of the study is the objective assessment of OD with a psychophysical test.

The major limitation of the study is none of the patients with COVID-19 anosmia had prior paranasal sinus CT or MRI to compare and evaluate the structural and mucosal effect of COVID-19 at OCs. Our postviral anosmia patient cohort had a limited percentage of patients with baseline and follow-up CT scans. With these limited data, we did not notice any significant change in OC width during follow-ups. Based on this observation, we are more in favor that the short interval between infection and OD is not adequate to cause the structural changes on CT. However, further follow-up of this patient cohort would clarify this topic. Also, the inability to measure the OS depths and the OBVs in MRI in the control group patients may be considered a limitation for the present study.

## Conclusion

In this study, patients diagnosed with COVID-19 who developed anosmia confirmed by psychophysical tests and patients who developed anosmia after viral upper respiratory tract infection had a larger OC compared to healthy controls. Larger OC width and volume may be a predisposing factor for viral upper respiratory tract infection and COVID-19–related anosmia. In addition, we have detected increased OC mucosal signal intensity in COVID-19 anosmia suggestive of inflammatory changes.
